# Long-term effect of anesthesia choice on patients with hepatocellular carcinoma undergoing open liver resection

**DOI:** 10.3389/fonc.2022.960299

**Published:** 2023-01-13

**Authors:** Runzhi Zhao, Xiyuan Xu, Li Sun, Guohua Zhang

**Affiliations:** ^1^ Department of Anesthesiology, National Cancer Center/National Clinical Research Center for Cancer/Cancer Hospital, Chinese Academy of Medical Sciences and Peking Union Medical College, Beijing, China; ^2^ Department of Anesthesiology, National Cancer Center/National Clinical Research Center for Cancer/Cancer Hospital & Shenzhen Hospital, Chinese Academy of Medical Sciences and Peking Union Medical College, Shenzhen, China; ^3^ Department of Anesthesiology, National Cancer Center/National Clinical Research Center for Cancer/Hebei Cancer Hospital, Chinese Academy of Medical Sciences, Langfang, China

**Keywords:** anesthesia, hepatocellular carcinoma, long-term outcome, propofol, inhalational anesthetics

## Abstract

Clinical and experimental evidence suggested that anesthesia choice can influence cancer progression and patients’ outcomes by modulating tumor microenvironment and tumorigenic pathways. Curative resection is the mainstay of therapy for hepatocellular carcinoma (HCC), which is an intractable disease due to high recurrence and poor prognosis. However, different anesthetics may play different roles in alleviating surgery-induced stress response and inflammatory cytokines release that are considered to be closely associated with proliferation, invasion and metastasis of tumor cells. Propofol, sevoflurane, non-steroidal anti-inflammatory drugs and local anesthetics have shown to exert anti-tumor effect on HCC mainly through regulating microRNAs or signaling pathways, while other inhalational agents, dexmedetomidine and opioids have the potential to promote tumor growth. In terms of anesthetic methods and analgesia strategies, propofol based total intravenous anesthesia and thoracic epidural analgesia could be preferred for HCC patients undergoing open liver resection rather than inhalational anesthesia. Local anesthesia techniques have great potential to attenuate perioperative stress response, hence they may contribute to more favorable outcomes. This review summarized the relations between different anesthesia choices and HCC patients’ long-term outcomes as well as their underlying mechanisms. Due to the complexity of molecules interactions and signaling pathways, further studies are warranted to confirm these results so as to optimize anesthesia strategy for HCC patients.

## Introduction

Anesthetic methods and agents can alter immune function and postoperative cancer recurrence (1, 2). There is a large body of literature suggesting the choice of anesthesia could exert either pro or anti-tumor effect on cancer patients (3). Growing evidence from animal and tumor cell line studies have suggested that volatile agents are more likely to enhance cancer progression through suppression of immune cell function and modulation of cancer cell signaling pathways (4–6). In contrast, propofol appears to suppress tumor growth and reduce the risk of migration *via* regulation of non-coding RNAs (e.g., microRNAs), signaling pathways and restoration of tumor microenvironment (7–9). Meanwhile, local anesthetics induce cancer cell apoptosis and reduce the methylation of tumor suppressor genes to hamper tumor cell proliferation (10, 11).

Hepatocellular carcinoma (HCC) is a lethal disease with poor prognosis and high recurrence rate (12). Although some noble therapies have constantly emerged in recent years, open liver resection has still been the safe choice for patients with risk factors, large tumors or with tumors located in the posterior segments of the liver (13). The postoperative analgesia approach for open hepatectomy seems to be less effective if the incision needs to be extended to the upper-level dermatomes of the abdomen. Insufficient analgesia can cause pulmonary dysfunction, especially with large incisions close to the diaphragm (14). In addition, postoperative coagulopathy is frequently seen following liver resection, hence the time to place and remove the epidural catheter should be within discretion of the anesthesia providers (15). Analgesic strategies for patients undergoing hepatectomy include epidural analgesia, peripheral nerve block (e.g., thoracic paravertebral block, quadratus lumborum block, transversus abdominis plane block, erector spinae plane block etc.) and local wound infiltration. Clinical evidence suggested the combination of epidural and general anesthesia could better attenuate immunosuppression related to stress response than general anesthesia and improve cancer patients’ outcomes (16).

This article reviewed the long-term effects (**Table 1**) and the relevant mechanisms (**Table 2**) of commonly used volatile, intravenous, local anesthetic agents and techniques as well as other anesthetic adjuncts (e.g. NSAIDS etc.) on HCC patients.

**Table 1:** The effects of anesthesia and analgesia choices on the prognosis of HCC patients.

Study Type	Cancer Type	Surgery	A versus B		Long-term outcomes (B compare to A)	References
			A	B		
Retrospective	Early- and intermediate-Stage HCC	Laparoscopic hepatic resection	INHA	Propofol	Lower recurrence rate	(17)
Retrospective	HCC	Hepatectomy	INHA (sevoflurane)	Propofol based TIVA	No difference in OS and RFS	(18)
Retrospective	HCC	Elective hepatectomy	INHA (Desflurane)	Propofol based TIVA	Better OS	(19)
Retrospective	Digestive Cancer (including HCC)	Hepatectomy	INHA	Propofol based TIVA	No difference in OS and RFS	(20)
Retrospective	HCC with PVTT	Aggressive hepatectomy	INHA (sevoflurane)	Propofol based TIVA	Better OS and RFS	(21)
Retrospective	HCC	HCC resection	EA with morphine	IA with fentanyl	Better long-term survival, no difference in RFS	(22)
Retrospective	HCC	Hepatectomy	PCIA with fentanyl	Parecoxib sodium +PCIA with fentanyl	Better DFS, no difference in OS	(23)
Retrospective	HCC	HCC resection	IA	EA	No difference in OS and RFS	(24)
Retrospective	HCC	Curative resection	GA	GA+EA	Better DFS and OS	(16)

Summary of studies comparing anesthesia and analgesia choices on HCC patients’ prognosis. Light color = anesthesia choices, dark color = analgesia choices; HCC, hepatocellular carcinoma; INHA, inhalational anesthesia; TIVA, total intravenous anesthesia; OS, overall survival; RSF, recurrence-free survival; PVTT, portal vein tumor thrombus; EA, epidural analgesia; IA, intravenous opioid analgesia; PCIA, patient-controlled intravenous analgesia; DFS: disease-free survival; GA, general anesthesia.

**Table 2:** The impact and mechanisms of different anesthesia agents on HCC cells.

Agents	Study type	Mechanisms	Functions					References
			Proliferation	Apoptosis	Invasion	Migration	EMT	
Sevoflurane	*in vitro*	miR-29a↑→Dnmt3a↓→PTEN↑→PI3K/AKT↓	↓	↑	↓	↓	–	(25)
		miR-29a-3p↑→CBX3↓	↓	↑	↓	↓	–	(26)
		miR25-3-p↓→PTEN↑→Akt/GSK-3β/β-catenin↓, c-Myc↓, MMP-9↓	↓	–	↓	↓	–	(27)
	*in vivo*/ *in vitro*	miR-148a-3p↑→ROCK1 ↓	↓	↑	↓	↓	–	(28)
Isoflurane	*in vitro*	SUMO2/3↑	↑	–	↑	↑	–	(29)
	*in vitro*	PI3K/AKT↓→NF-κB↓	↓	↑	↓	↓	–	(30)
Propofol	*in vitro*	miR-199a↑→MMP-9↓	–	–	↓	–	–	(31)
		miR-199a↑→caspase-8,9↑	–	↑	–	–	–	(32)
		miR-134↑→BCL-2↓, cleaved caspase-3↑	–	↑	–	–	–	(33)
		NORAD↓→miR-556-3p↓→MIEN1↓	↓	–	–	–	↓	(34)
		miR-105↑→JAK2/STAT3↓	↓	↑	–	–	–	(35)
		NET1↓→ERK/VEGF↓	↓	–	↓	↓	–	(8)
		HMGA2↓→Wnt/β-catenin↓	↓	↑	↓	–	–	(36)
		Twist1↓→MMP-2, MMP-9↓, E-cadherin↑	↓	–	↓	↓	–	(37)
	*in vivo*/ *in vitro*	miR-219-5p↑→glypican-3/Wnt/β-catenin↓	↓	–	↓	↓	↓	(38)
		lncRNA HOXA11-AS↓→miR-4458↑	↓	↑	↓	↓	–	(39)
		lncRNA H19↓→miR-520a-3p↑→LIMK1↓	↓	↑	↓	↓	–	(40)
	*in vivo*	TAMs↑→MVs↑→miR-142-3↑→RAC1↓	↓	–	↓	↓	–	(41)
		MMP-2, VEGF↓	↓	–	–	–	–	(42)
		AMPK↑→autophagy	↓	↑	–	–	–	(9)
Dexmedetomidine	*in vitro*	miR-130a↑→EGR1↓	↓	↑	↓	↓	–	(43)
	*in vivo*	IL-6 released by aHSCs↑, STAT3↑	↑	–	–	↑	–	(44)
Morphine	*in vivo*/ *in vitro*	MMP-9, uPA↓	↓	↑	↓	↓	–	(45)
		DDX49↓→MAPK↓	↓	↑	↓	↓	–	(46)
	*in vivo*/ *in vitro*	PI3K/Akt/HIF-1α↑→VEGFA↑	↑	–	–	↑	–	(47)
Celecoxib	*in vitro*	COX-2-PGE2-EP2-p-Akt/p-ERK↓→E-cadherin↑	↓	↑	↓	↓	–	(48)
	*in vivo*/ *in vitro*	PNO1↓→AKT/mTOR↓	↓	–	–	↓	–	(49)
	*in vivo*	NK cytotoxicity suppression↓	–	–	–	↓	–	(50)
Midazolam	*in vivo*/ *in vitro*	miRNA-124-3p↑→PIM-1↓	↓	↑	↓	↓	–	(51)
	*in vitro*	miR-217↑	↓	↑	↓	↓	–	(52)
Lidocaine	*in vivo*/ *in vitro*	ERK1/2 and p38↑→cleaved caspase-3 and Bax↑, BCL-2↓	↓	↑	–	–	–	(10)
		circ_ITCH/miR-421/CPEB3↑	↓	↑	↓	↓	–	(53)
	*in vitro*	USP14↓→PI3K/Akt↓	↓	↑	↓	↓	↓	(54)
Bupivacaine	*in vivo*/ *in vitro*	PI3K/Akt↓, MAPK↓	↓	↑	↓	↓	–	(55)
Ropivacaine	*in vitro*	damaged mitochondria; caspase-3 ↑	↓	↑	–	↓	–	(56)
	*in vivo*/ *in vitro*	IGF-1R↓→PI3K/AKT/mTOR ↓	↓	↑	↓	↓	↓	(57)
Amide-type LA	*in vitro*	RASSF1A expression↑	↓	–	–	–	–	(11)
Procaine	*in vivo*/ *in vitro*	demethylation	↓	–	–	–	–	(58)

Summary of studies about the impact and mechanisms of different anesthesia agents on HCC cells. Light color = anti-tumor effect, dark color = pro-tumor effect; —, indeterminate or limited data; ↑, increase; ↓, decrease; EMT, epithelial-mesenchymal transition; miR, microRNAs; Dnmt3a, DNA methyltransferase 3α; PTEN, phosphatase and tensin homolog; PI3K, phosphatidylinositol 3 kinase; AKT, protein kinase B; CBX3, chromebox protein homolog 3; GSK-3β, glycogen synthase kinase 3β; MMP, metaloproteinases; ROCK1, Rho-associated protein kinase 1; SUMO2/3, small ubiquitin-like modifier 2 and 3; NF-κB, nuclear factor κB; BCL-2, B cell lymphoma-2; lncRNAs, long non-coding RNAs; HOXA11-AS, HOMEOBOX A11 antisense RNA; LIMK1, LIM domain kinase 1; NORAD, non-coding RNA activated by DNA damage; MIEN1, migration and invasion enhancer 1; TAMs, tumor-associated macrophages; MVs, microvesicles; RAC1, ras-related C3 botulinum toxin substrate 1; NET1, neuroepithelial cell transforming gene 1; ERK, extracellular signal-regulated kinase; VEGF, vascular endothelial growth factor; HMGA2, High mobility group A2; AMPK, adenosine monophosphate-activated protein kinase; EGR1, early growth response 1; aHSCs, activated hepatic stellate cells; DDX49, DEAD-box helicase 49; MAPK, mitogen-activated protein kinases; HIF-1α, hypoxia-inducible factor-1α; VEGFA, vascular endothelial growth factor A; COX-2, cyclooxygenase-2; PGE2, prostaglandin E2; EP2, PGE2 receptor 2; PNO1, partner of NOB1; mTOR, rapamycin; NK cell, natural killer cell; USP14, ubiquitin specific peptidase 14; CPEB3, cytoplasmic polyadenylation element binding protein 3; IGF-1R, insulin-like growth factor-1 receptor; LA, local anesthetics; RASSF1A, ras association domain family 1A.

## Volatile anesthetic agents

Inhalational anesthetics like isoflurane, desflurane, halothane have already been proved to reduce the number of natural killer (NK) cells or NK cell tumor cytotoxicity (4, 59), which are closely involved in cancer immunosurveillance and immunoediting (60). Isoflurane and sevoflurane also suppressed the release of interleukin-1β (IL-1β) and tumour necrosis factor α (TNF-α) stimulated by tumor cells in peripheral blood mononuclear cells (PBMCs), thus promoting tumorigenesis (61). A retrospective study indicated the inhalational anesthetics were associated with higher recurrence rate compared with propofol in HCC patients receiving laparoscopic hepatic resection (17).

### Sevoflurane

The influence of sevoflurane on cancer is controversial since it has both anti-tumor and pro-tumor effects (62, 63). MicroRNA (miR)-29a, a potential prognostic biomarker, was down-regulated in HCC patients according to the previous studies (64). The exposure of sevoflurane could restore the level of miR-29a and inhibit tumor progression by lowering the expression of long non-coding RNA KCNQ1 opposite strand/antisense transcript 1 (lncRNA KCNQ1OT1) (26), which led to the reduction of DNA methyltransferase 3 alpha (Dnmt3a) (25) and chromebox protein homolog 3 (CBX3) (26) in HCC cell lines. *In vitro* studies also suggested that sevoflurane-stimulated tumor cells exhibited higher level of phosphatase and tensin homologue (PTEN) and inactivation of phosphatidylinositol 3 kinase (PI3K)/protein kinase B (Akt) signaling pathway (25, 27), a crucial signaling in tumorigenesis (65). Also, in both cell lines and animal models experiments, researchers observed a correlation of increased level of miR-148-3p and decreased Rho-associated protein kinase 1 (ROCK1) after sevoflurane treatment (28), which might account for its antitumor property.

In order to further explore the long term cancer-related outcomes sevoflurane could exert, researchers compared the overall mortality and recurrence-free survival rates of HCC patients in a retrospective cohort, and concluded that there was no difference between propofol based total intravenous anesthesia (TIVA) and sevoflurane anesthesia (18). A meta-analysis study also adequately evidenced that the use of sevoflurane might not exert any unfavorable effect on cancer patients’ overall survival (66). However, recently a study on HCC patients with portal vein tumor thrombus (PVTT) showed that sevoflurane based inhalation anesthesia was related to worse clinical outcomes comparing TIVA (21). Unlike other clincal trials, patients in this cohort had larger tumor burden (87% >10cm), and the distinction between TIVA and sevoflurane anesthesia was amplified by the severity of the disease according to its subgroup analysis.

### Isoflurane

In Hep-3B cell line, short-term isoflurane exposure (<12h) might facilitate cancer cell invasion by enhancing the formation of small ubiquitin-like modifier 2 and 3 (SUMO2/3) conjugates, a kind of stress indicators, while the inhibitor of SUMO2/3 could reverse the response (29). But in primary cultured cells from patients undergoing hepatectomy and general anesthesia, evidence indicated that isoflurane could reduce HCC aggressiveness *via* down-regulating PI3K/AKT signaling pathway mediated nuclear factor kappaB (NF-κB) activity (30). These conflicts could be attributed to the different cell lines and distinctive time of isoflurane exposure (12h versus 24-72h).

### Desflurane

Studies showed that desflurane did not affect NK cell counts and was in favor of preserving the ratio of CD4+/CD8+ T cells in breast cancer surgery (67, 68). However, most related studies considered desflurane to be associated with worse outcomes (3, 69). As for HCC patients, clinical evidence showed that the use of desflurane might lead to lower overall survival and higher recurrence rates compared with propofol (19).

To draw a conclusion from the available laboratory and clinical studies, among volatile anesthetic agents, sevoflurane showed anti-tumor effects in most cases, desflurane was more likely to exhibit pro-tumor effects, while isoflurane presented a paradoxical effect on different tumor cell lines. Other inhalational anesthetic agents were seldom studied regarding their long-term effects on HCC patients.

## Intravenous anesthetic agents

### Propofol

There is increasing evidence suggesting that propofol can inhibit proliferation, metastasis, and induce apoptosis of malignant tumors, thereby influencing the prognosis of cancer patients (7, 31). As of today, the underlying mechanism of its anti-tumor effect has not been fully understood, but it may relate to the regulation of non-coding RNAs (e.g., miRNAs, lnc RNAs), alteration of signaling pathways, as well as restoration of microenvironment.

Propofol can modulate miRNAs expressions and exert inhibitory effect on many carcinoma-crucial pathways linked to proliferation, migration, and apoptosis (7). An *in vitro* study showed that propofol could up-regulate miR-199a to inhibit the adhesion of HCC *via* downregulating metaloproteinases-9 (MMP-9) expression, an enzyme that could degrade extracellular matrix (ECM) proteins and mediate cancer metastasis (31). Also, activation of caspase-8 and caspase-9 mediated by increased level of miR-199a might be the underlying mechanism accounting for propofol-induced HCC cell apoptosis (32). Similarly, propofol appeared to up-regulate miR-134 expression, inhibit B cell lymphoma-2 (BCL-2) expression level, and elevate cleaved caspase-3 level, thereby inducing apoptosis as well (33). Propofol also increased the expression of miR-219-5p, a tumor suppressor candidate, which directly inhibited the glypican-3 mediated activation of Wnt/β-catenin pathway, and subsequently regulating the progression, metastasis and epithelial-mesenchymal transition (EMT) of HCC (38). LncRNAs are mRNA-like transcripts ranging from 200 nucleotides to 100 kilobases. It has been proved that some lncRNAs can modulate the expression of miRNAs so that they act as an axis and can be influenced by propofol at any level. For example, propofol could inhibit HCC progression by increasing the level of miR-4458 *via* modulating HOMEOBOX A11 antisense RNA (HOXA11-AS)/miR-4458 (39) axis as well as reducing HCC growth and metastasis through lncRNA H19/miR-520a-3p/LIM domain kinase 1 (LIMK1) axis (40). Apart from that, miR-556-3p played an essential role in propofol regulated non-coding RNA activated by DNA damage (NORAD)/miR-556-3p/migration and invasion enhancer 1 (MIEN1) axis, which involved in suppressing HCC cell proliferation and EMT progression (34). In addition, propofol increased the expression of miR-105, a potential JAK2/STAT3 signaling inhibitor, serving as a key mediator in HCC apoptosis process (35).

Evidence shows that some signaling pathways may play critical roles in liver cancer progression, and researchers have explored how propofol regulates tumor behaviors through these signaling pathways. Extracellular signal-regulated kinase 1/2 (ERK1/2) are protein-serine/threonine kinases that participate in the Ras-Raf-MEK-ERK signal transduction cascade, which are activated in one third of human cancers (70). A pathological analysis of HCC samplings showed that high p-ERK1/2 levels might be associated with elevated HCC recurrence and worse overall survival (71). In Fei G et al’s study, the levels of p-ERK1/2 were decreased in HCC cell lines treated with propofol, leading to less aggressive growth and invasion behaviors (8). Down-regulation of p-ERK1/2 might be caused by neuroepithelial cell transforming gene 1 (NET1), a new member of the tetraspanins group and a therapeutic potential target for cancer (72). High mobility group A2 (HMGA2) is an architectural transcriptional regulator that could independently predict the prognosis of HCC patients (73). And in Ou W et al’s study, propofol decreased the level of HMGA2, and subsequently attenuated the activity of Wnt/β-catenin pathway, thereby inducing apoptosis and inhibiting HCC proliferation as well as invasion (36). In fact, signaling pathways like nuclear factor E2-related factor-2 (Nrf2), NF-κB are well-illustrated in the relations between propofol and other kinds of cancers. However, as of yet there is little literature about the direct link between these pathways and the effect of propofol on HCC biological behaviors till now.

**Figure 1:**
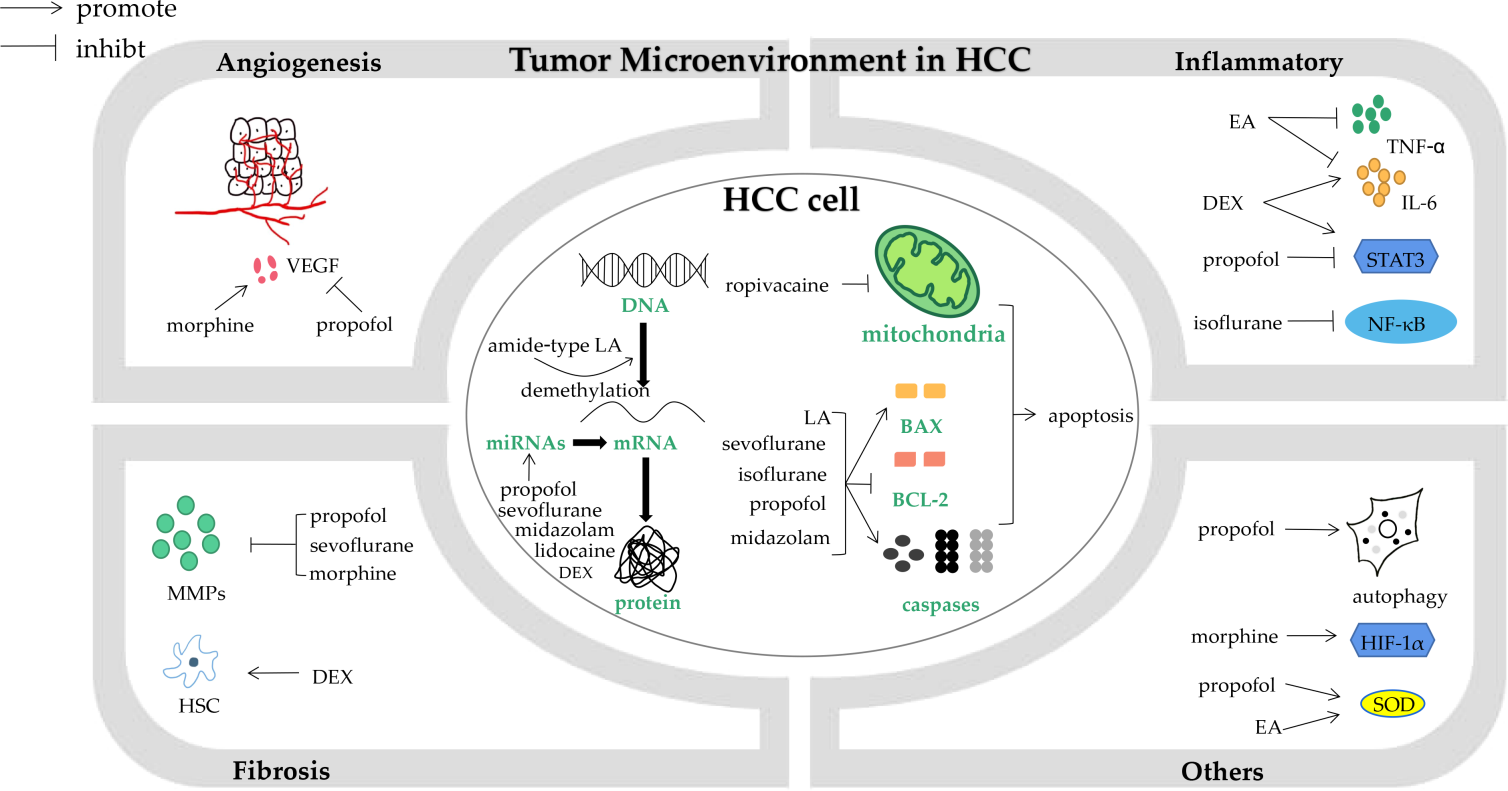
The impact of anesthesia agents and techniques on biological processes in hepatocellular carcinoma cells and tumor microenvironment. HCC, hepatocellular carcinoma; VEGF, vascular endothelial growth factor; MMPs, metaloproteinases; HSC, hepatic stellate cells; DEX, dexmedetomidine; LA, local anesthetics; miRNAs, microRNAs; BCL-2, B cell lymphoma-2; EA, epidural analgesia; TNF-α, tumour necrosis factor α; IL-6, interleukin-6; NF-κB, nuclear factor κB; HIF-1α, hypoxia-inducible factor-1α; SOD, superoxide dismutase.

The tumor microenvironment (TME) is a mixture of tumoral cells within the ECM, surrounded by a complex mix of stromal cells and the proteins they secrete. The biological processes involved in TME include angiogenesis, inflammation, fibrosis and other processes like hypoxia, oxidative stress and autophagy (**Figure 1**) (74). Propofol can affect TME in different ways. Vascular endothelial growth factor (VEGF), the most important factor in angiogenesis, could be inhibited by propofol in HCC patients *via* down-regulating NET1 (8). An experiment examined oxidative stress caused by H2O2, and found that in contrast with pentobarbital, propofol exerted a protective effect on hepatocytes under oxidative stress (75), it is also the case for what had discovered before when comparing propofol to etomidate (76). There was a meta-analysis found that propofol might also have a beneficial impact on HCC patients after liver ischemia reperfusion injury (LIRI), demonstrated by a lower level of malondialdehyde (MDA), alanine transaminase (ALT), aspartate transaminase (AST) and a higher level of superoxide dismutase (SOD) (77). Propofol could also lead to down-regulation of MMPs and increase of E-cadherin by impairing Twist1 expression, and inhibit HCC proliferation, migration, and invasion as a result (37).

*In vivo* studies provide a holistic perspective of the effect propofol has on HCC. Studies had observed that propofol treatment significantly decreased the tumor volume and slowed proliferation in nude mice model (38), and even in a propofol dose-dependent manner (78). Besides, by constructing Over H19‐Propofol‐Huh7‐exo mouse model, researchers found the high level exsomal lncRNA H19 promote the growth and metastasis of HCC tumors, hence verifying propofol’s anti-tumor effect conversely (40). Also, in tumor-bearing mice models, researchers found that propofol activated tumor-associated macrophages (TAMs) were able to secrete microvesicles (MVs), which delivered extrinsic miR-142-3p to HCC cells, thereby down-regulating ras-related C3 botulinum toxin substrate 1(RAC1) expression and inhibiting HCC invasion (41). In terms of tumor microenvironment, a mice model experiment proved that propofol could decrease levels of MMP-2 and VEGF in a dose-dependent manner (42), which reduced the chance of cancer metastasis. Studies in xenograft mice tumor models also showed autophagy, a catalytic process related to cancer initiation and progression, could be induced by propofol *via* activating adenosine monophosphate-activated protein kinase (AMPK) pathway (9).

Clinical evidence is also in correspondence with the above results. A retrospective study, which compared the recurrence of early- and intermediate-stage HCC patients after hepatic resection, revealed that the use of propofol as the main anesthetic agent might be superior to inhalational anesthetics in terms of recurrence rate (17). Similarly, Lai and his colleague also found the overall mortality and distant metastasis rate were higher in desflurane group than in propofol based TIVA group (19). Meanwhile, a nationwide retrospective cohort in Japan suggested the overall survival and recurrence-free survival rates were not significantly different between volatile anesthesia and propofol based TIVA groups of patients with digestive tract cancers including HCC (20), which was consistent with that of a Chinese cohort (18). Through combining existing clinical evidence, Chang et al. conducted a meta-analysis and concluded better overall survival and recurrence-free survival for patients with hepatobiliary cancer under propofol-based TIVA (66).

### Dexmedetomidine

Dexmedetomidine (DEX), a centrally acting alpha-2 agonist, plays an essential role in perioperative period as it can provide safe sedation, reduce opioids consumption and stabilize hemodynamic status. Clinical evidence indicated that DEX’s central effect reduced the release of norepinephrine and thereby inhibiting inflammatory response. A meta-analysis showed the administration of DEX during perioperative period lowered the levels of catecholamine, cortisol, glucose, IL-6, TNF-α, and C reactive protein (CRP), whereas increased the expression of NK cells, B cells, CD4+T cells, and the ratios of CD4+/CD8+ and Th1/Th2 (79). This inflammatory attenuation may show great anti-tumor effect in liver cancer patients since the inflammatory process provides a pro-tumorigenesis microenvironment. An *in vitro* study in HCC indicated that DEX could suppress HCC cell proliferation and induce apoptosis by down-regulating miR-130a and inhibiting early growth response 1 (EGR1) expression (43). However, in a fibrosis mouse model experiment, it proved that DEX might not induce the pathogenesis of HCC cell itself. Instead, it promoted HCC progression by activating hepatic stellate cells *via* ARD2A-induced IL-6 secretion and STAT3 activation (44).

### Etomidate

Etomidate is commonly used for elder patients and those with unstable hemodynamic status. Comparing with volatile anesthetics, etomidate shows less inhibition to NK cell-mediated cytotoxicity (59). The treatment of etomidate significantly mitigated lipopolysaccharide (LPS)-induced TNF-α and IL-6 levels and activation of NF-kB signaling pathway in macrophages (80). As for its effect on tumor, studies conducted in lung cancer illustrated that etomidate could curb cancer cell proliferation and invasion through different mechanisms (81, 82).

### Ketamine

Ketamine is long believed to affect the immunoregulatory activities of immune cells. It inhibits the maturation of bone marrow-derived dendritic cells (BMDCs) and hampers the ability of dendritic cells (DCs) to prime a Th1-biased immune response (83). NK cell function is largely related to tumor recurrence and mortality (84). In a mouse model experiment using breast cancer cell line, ketamine reduced the number of NK cells and improved metastasis drastically compared with other anesthetics (4). However, Beilin B et al.’s results showed a small dose of ketamine before induction might prevent immune function from inflammatory cascade by inhibiting the release of IL-6 and TNF-α in early postoperative period (85). In recent years, ketamine has been proved not to change natural killer cell cytotoxicity in cancer patients and may even inhibit cancer cell growth (86–88).

## Perioperative adjuvant medications

### Opioids

Opioids, such as morphine, are widely used to alleviate pain perioperatively, which is generally thought to be immunosuppressive. Opioids exert restraining effect on innate immunity by suppressing NK cell cytotoxicity, inhibiting macrophage and neutrophil phagocytosis, and decreasing cytokine production (89). However, opioids also play an imperative role in relieving postoperative pain, which is a potential risk factor of tumor metastasis and morality (90).

Morphine, a generally studied opiate, has been shown to have both anti- and pro-metastasis potential in HCC. In an *in vitro* study, HCC cell lines pre-exposed to morphine did not increase the proliferation, instead they showed a less progressive ability of sphere formation and were prone to apoptosis *via* down-regulating MMP-9 and uPA, which are both ECM-degrading enzymes (45). In a nude mouse model, morphine also reduced lung colonization (45). Another study showed morphine could down-regulate DEAD-box helicase 49 (DDX49) expression and mitogen-activated protein kinases (MAPK) signaling to exert anti-tumor effect (46). On the contrary, morphine was also proved to promote angiogenesis by activating PI3K/Akt/HIF-1α pathway and increasing secretion of VEGFA in HCC (47). Sufentanil is also commonly used during perioperative period. A randomized animal study indicated that sufentanil-based postoperative analgesia could increase Th17 cells and FoxP3+ regulatory T (Treg) cells, but no difference in 5-year mortality was observed (91). A retrospective study pertaining to the choice of postoperative analgesia gave us information that the use of epidural analgesia with morphine might have risk of increasing cancer recurrence and death in contrast to intravenous analgesia with fentanyl (22). The heterogeneous nature of these studies can be attributed to different cell lines and morphine concentrations. Therefore, more studies with same standards are needed to clarify whether opioids could exert anti-tumor effect on HCC or not.

### Non-steroidal anti-inflammatory drugs

Non-steroidal anti-inflammatory drugs (NSAIDs) inhibit cyclooxygenase (COX) enzymes, which can catalyze the synthesis of prostaglandins (PGs) in inflammatory processes. Clinical data analysis showed that the COX-2 expression in HCC tumor sample was closely associated with TNM stage, tumor size, lymphovascular invasion and distant metastasis, and 5-year survival rate (48). *In vitro* experiments suggested celecoxib treatment could up-regulate E-cadherin and inhibit COX-2-PGE2-PGE2 receptor 2 (EP2) -p-Akt/p-ERK pathway, and subsequently inhibit cell proliferation, induce apoptosis, and constrain migration and invasion (48). Recently, a study focused on the mechanisms other than COX-2 found that celecoxib’s anti-tumor effect on HCC might also involve partner of NOB1 (PNO1) and AKT/rapamycin (mTOR) signaling pathway (49). In an animal model, perioperative administration of COX-2 inhibitor could attenuate the inflammatory stress caused by surgery and reduce the risk of tumor metastasis (50). Parecoxib sodium combined with fentanyl for PCIA provided adequate analgesia, preserved immune function, and postponed HCC recurrence more favorably than fentanyl alone (23). Apart from its use for anesthesia, it has been proved by a meta-analysis that long-term NASIDs use could prevent the development of HCC (92), and reduce the recurrence rate of patients at risk after curative resection (93).

### Benzodiazepines

Several meta-analysis articles suggested that the use of benzodiazepines might not be associated with cancer mortality (94, 95). But preclinical experiment in HCC cells showed that midazolam increased the level of miR-124-3p and consequently repressed the expression of PIM-1, a survival kinase, leading to cell cycle arrest and apoptosis (51). After treated with midazolam, HCC cell viability, migration were inhibited and apoptosis rated was enhanced in accordance with the increasing level of miR-217 (52).

## Regional anesthesia

Since the regional nerve block techniques gain popularity in these years, the perioperative administration of local anesthetics (LA) has been widely employed, especially for cancer patients. Numerous studies and experiments have suggested that LA could improve postoperative rehabilitation and reduce cancer recurrence (96, 97).

### Local anesthetics

The commonly used LA are amide-type, such as lidocaine, bupivacaine, and ropivacaine, which can impede the release of inflammatory mediators and down-regulate the activation of immune cells, thereby suppressing immune and inflammatory response (98). And the analgesic characteristic of LA presented an anti-tumor effect by inducing the consumption of opioids through effectively alleviating the pain of surgery and cancer (99). In addition to these indirect action, LA can exert inhibitory effect on tumor progression directly. It has been well accepted that LA can cause apoptosis in cancer cells. In HCC cell lines, Xing W et al.’s study had seen significantly increased apoptosis rate in cells treated with lidocaine. They also proved this might be the result of activation of ERK1/2 and p38, and then the activation increased cleaved caspase-3 and Bax levels and decreased BCL-2 level (10). The similar pro-apoptotic effect has been observed with bupivacaine as well (55). Another study validated ropivacaine’s effect on caspase-3, and proposed that ropivacaine could damage the structure of mitochondria and interfere its function, then lead to cell death (56). Furthermore, LA can also inhibit HCC cell proliferation, migration, invasion by regulation of signaling pathways. PI3K/AKT/mTOR signaling pathway is usually activated in HCC patients and closely related to the anti-tumor effect of LA (100). Lidocaine down-regulated PI3K/Akt pathway *via* the reduction of ubiquitin specific peptidase 14 (USP14) level, leading to lower-level proliferation and invasion capacity (54). Similarly, ropivacaine and bupivacaine both could suppress PI3K/Akt pathway and reduce tumor cell proliferation and metastasis (55, 57). Other pathways like MAPK can also be down-regulated with LA treatment and involved in HCC progression (55).

Transcriptional silencing of tumor suppressor genes through methylation of their promoters could lead to tumorigenesis (101). Tumor suppressive gene ras association domain family 1A (RASSF1A) has been shown to be silenced by promoter methylation in HCC according to previous findings (102). Dongtai Chen and his colleagues evaluated methylation levels of RASSF1A in HCC cell lines treated by amide-type LA and discovered a decreased level of methylation and increased expression of RASSF1A both in mRNA and protein levels, followed by reduced cell proliferation (11). This demethylating effect of LA on hepatoma cells was firstly discovered in 2007 in a study concerning procaine, an esters-type agent (58). In addition, the increased level of cytoplasmic polyadenylation element binding protein 3 (CPEB3) induced by lidocaine might also be a part of LA’s anti-tumor mechanisms, which could be regulated by circ_ITCH/miR-421/CPEB3 axis (53).

### Local anesthesia techniques

Thoracic epidural block (TEA) is a historically classic pain-relief method, which was strongly recommended by American Pain Society (103). TEA combined with general anesthesia (GA) could provide elder HCC patients with better cognitive status, less release of inflammatory factors, such as IL-6, IL-1, TNF-α, and lower stress response than GA alone (104, 105). Application of TEA was associated with less activation of hypothalamic-pituitary-adrenal (HPA) axis, better preservation of the Th1/Th2 balance and maintenance of CD3+, CD4+, and CD4+/CD8+ T cells, indicating an immune protective effect on HCC patients under surgery (106–108).

Clinical trials have been conducted to clarify whether TEA combined with GA could exert anti-tumor effect on HCC patients during surgery. A retrospective cohort recruiting 772 patients undergoing selective curative resection surgery suggested that GA combining with TEA showed higher 3-year overall survival and disease-free survival rates, rendering GA solely as the independent predictors of poor survival and high risk of recurrence, respectively (16). But another single-center study using electronic medical records found that TEA did not have a definite relation with the recurrence-free survival and overall survival of HCC patients (24). These two retrospective cohorts were based on patients with different baseline characteristics. Patients in the latter were with higher AFP level, larger tumor size, and longer anesthesia time, and the follow-up time was longer than the former. Therefore, with the intrinsic retrospective limitations, it is a call for future randomized controlled trial to be conducted.

Other regional anesthesia techniques like transverse abdominal plane block (TAPB), thoracic paravertebral blocks (TPVBs), quadratus lumborum block (QLB), erector spinae plane block (ESPB) and local wound infiltration have been reported to be used as analgesic methods for HCC patients undergoing open liver resection, but there is devoid of evidence concerning the relationship between these techniques and long-term prognosis. An analysis of ACS NSQIP database regarding the one-month mortality and overall morbidity rates of patients under hepatectomy revealed that the use of regional abdominal wall nerve block (mostly TAPB) was associated with better short-term morbidity like shorter duration of hospital stay compared with TEA, while there was no significant difference pertaining to overall morality rates (109).

## Conclusions

New techniques, drugs and regimens are constantly explored and tentatively conducted in clinical practices, especially for cancer patients. The perioperative factors linked with recurrence are the circulating tumor cells and minimal residual disease. Therefore, it is pivotal for us anesthesiologists to maintain the homeostasis and preserve patients’ immunity in this period. Despite there is no conclusive evidence to provide a clear protocol, there are some beneficial actions we can take. Firstly, propofol-based TIVA should be routinely used if possible for HCC patients undergoing open hepatectomy, if not, sevoflurane-based inhalational anesthesia ought to be the alternative. Next, local anesthesia agents and techniques are recommended to implement, especially for patients contraindicated to epidural anesthesia. Because local anesthesia shows great superiority in postoperative analgesia and attenuation of inflammatory response. Hence, the ability to skillful use the ultrasound should be added to the anesthesia education system. Furthermore, in accordance with enhanced recovery after surgery (ERAS) program, the effective multimodal analgesic methods including opioids and NSAIDS are indispensable. Undoubtedly, a clear anesthesia protocol for HCC patients far from well established, more prospective RCTs and multicenter studies on cancer recurrence are warranted for more scientific and convinced results.

## Author contributions

RZ and XX wrote the manuscript. LS and GZ revised and corrected the manuscript. All authors read and approved the final manuscript.
